# Methodological issues in the designing and reporting of frequentist and Bayesian meta‐analysis to assess COVID‐19 outcomes among PLHIV with various comorbidities

**DOI:** 10.1002/jia2.25946

**Published:** 2022-06-09

**Authors:** Ram Bajpai, Vivek Verma, Gyan Prakash Singh

**Affiliations:** ^1^ School of Medicine Keele University Staffordshire UK; ^2^ Department of Statistics Assam University Silchar India; ^3^ Department of Statistics Banaras Hindu University Varanasi India

**Keywords:** Methodological quality, Meta‐analysis, Bayesian methods, Evidence synthesis, PRISMA reporting guidelines, COVID‐19

1

Dear Editor,

We read the recent article with great interest by Wang and Jonas [1], who assessed the likelihood of severe COVID‐19 outcomes among people living with HIV/AIDS (PLHIV) with various comorbidities using both frequentist and Bayesian meta‐analysis approaches. Findings from this systematic review are important for PLHIV with coexisting diabetes, hypertension, cardiovascular disease, respiratory disease and chronic kidney disease as they are at a higher risk of developing severe COVID‐19 outcomes. However, we identified several methodological issues related to planning, conduct, and analytical methods and its reporting that limits the acceptability and generalizability of the findings from this study and could mislead in clinical decision making.

Authors should have used the Preferred Reporting Items for Systematic reviews and Meta‐Analyses (PRISMA) 2020 [2] for reporting and presenting their flow diagram instead of using the PRISMA extension for network meta‐analysis. Similarly, authors did not report the International Prospective Register of Systematic Reviews registration information of this systematic review, which is essential to avoid duplication and transparency. Without a registered protocol, we will never know about what was planned and what has been presented. These are essential features when you conceptualize and report a systematic review. Such practice questions the integrity of overall conduct and reliability of findings. Further, no information about the model selection (i.e. fixed or random) was provided for frequentist meta‐analysis, and both were estimated for Bayesian meta‐analysis. However, fixed or random effect model must be selected as a priori based on the differences in the way studies were conducted and study characteristics [3]. Authors did not present frequentist meta‐analysis results with the prediction interval, which is a common practice to allow more informative inferences in meta‐analyses [4, 5]. The prediction interval reflects the expected range of true effects in similar future studies over different settings. Authors have wrongly planned and tested (using meta‐regression which is suitable to explore heterogeneity in estimates) publication bias, which will mislead to readers. Publication bias should not be assessed when included studies are less than 10 in a meta‐analysis due to low power as recommended in the literature [6].

Both frequentist and Bayesian methods have been applied to generate effect estimates, which is unusual considering both methods to generate same effect estimates as they are fundamentally different in their nature [7]. Authors discussed meta‐analysis under two different setups, viz. frequentist and Bayesian perspective techniques, but the reasoning being using both complementary methodologies, comparison and evaluation of the performances can be more elaborated for better clarity to readers. Authors have used the prior predictive distribution for sensitivity analysis and based on that, they selected half‐normal distribution for further analysis. But prior predictive distribution is considered in the model before taking the observation and is very sensitive to the choice of prior [8]. In order to assess the performance of the prior in association of the given data, one should use the posterior predictive probability [9]. The most appropriate criteria for model selection will be Bayes factor [10]. The inferential procedure presented in the manuscript needs more elaboration on the specification and selection of priors as well as the suitability of the appropriate model. Authors are also lacking on the several aspects of reporting in the methods and results (such as Markov chain Monte Carlo (MCMC) chain convergence and resolution, model diagnostics and reproducible codes) of a Bayesian analysis as recommended in the literature, which is essential for transparency and robustness [11].

We believe that authors should address the points raised and the overall purpose of the presented points, this will only improve the conduct and reporting of frequentist and Bayesian methods in meta‐analysis to benefit researchers at large.

Sincerely,

Ram Bajpai

Vivek Verma

Gyan Prakash Singh

## COMPETING INTERESTS

The authors have no competing interests to declare.

## AUTHORS’ CONTRIBUTIONS

RB, VV and GPS drafted, read and reviewed the final version of letter.
